# Supplementary material for: The Change of Lumbar Spinal Stenosis Symptoms over a Six-Year Period in Community-Dwelling People
Source: Medicina (Kaunas). 2021 Oct 16;57(10):1116. doi: 10.3390/medicina57101116 (PMC8537511; doi:10.3390/medicina57101116)
Supplement: Supplementary file 1 [file medicina-57-01116-s001.zip › medicina-1383420-supplementary.pdf]

Fig. 1S. Time course of LSS-symptom for six-year duration; Subjects without either knee or hip OA in 2004

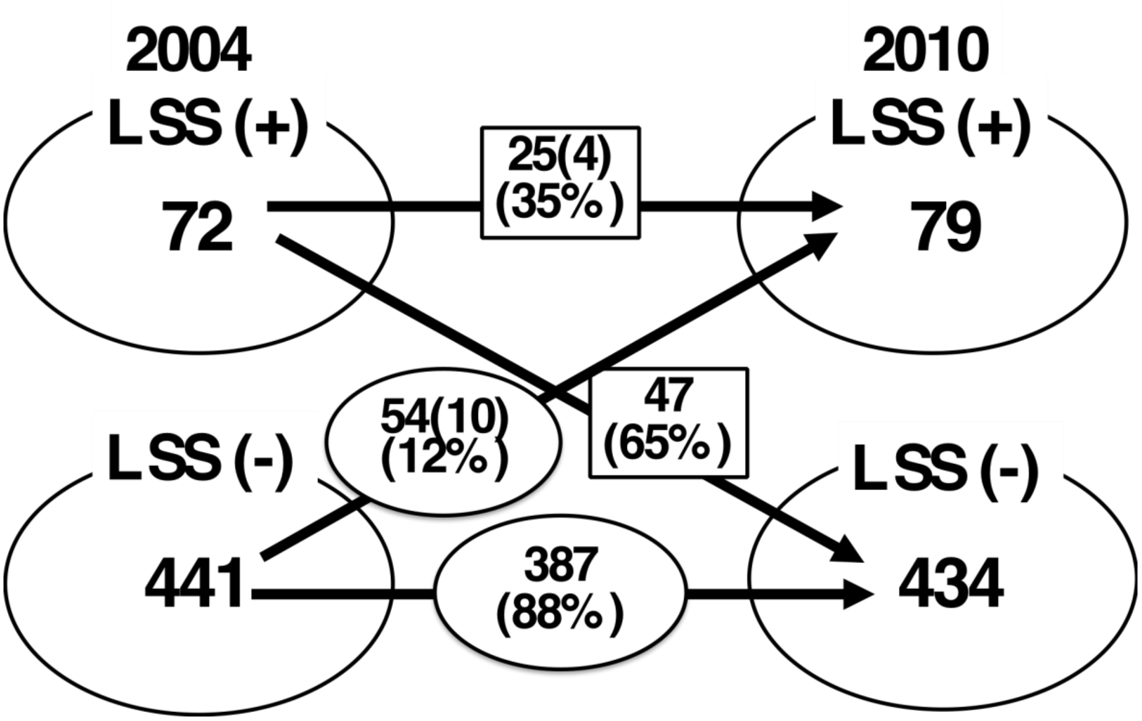

( ) : Number of operation case

Table S1. Change of LSS-symptoms positive and LSS-symptoms negative groups by age; Subjects without either knee or hip or OA

Age in 2004	Change of LSS symptoms (*n* = 513)					
	LSS (+) in 2004 → 2010			LSS (-) in 2004 → 2010		
	(+) → (+)	(+) → (-)	*n*	(-) → (+)	(-) → (-)	*n*
40–49 y	1 (33.3)	2 (6.7)	3	2 (6.3)	30 (93.8)	32
50–59 y	5 (55.6)	4 (44.4)	9	7 (9.5)	67 (90.5)	74
60–69 y	9 (34.6)	17 (65.4)	26	20 (10.4)	172 (89.6)	192
70–79 y	10 (29.4)	24 (70.6)	34	25 (17.5)	118 (82.5)	143

Abbreviations: LSS, lumbar spinal stenosis

Table S2. Change in RDQ scores of the subjects with/without LSS symptoms; Subjects without either knee or hip OA in 2004

Change of LSS symptoms (*n* =509)	2004	2010	p value
(+)→(+)	46.6 ± 7.9[(1)]	48.4 ± 7.9	0.3942
(+)→(-)	49.8 ± 6.8[(1)]	53.5 ± 7.4	0.0520
(-)→(+)	55.3 ± 6.5	52.4 ± 8.9 [(2)]	0.1334
(-)→(-)	56.4 ± 5.3	57.1 ± 6.3[(2)]	<0.0001

Average ± SD (1); $p < 0.05$, (2); $p < 0.0001$

Abbreviations: LSS, lumbar spinal stenosis; RDQ, Roland-Morris Disability Questionnaire; SD, standard deviation

Table S3. Changes in eight domains of SF-36 of the subjects with/without LSS symptom; Subjects without either knee or hip OA in 2004

	Time course of LSS (*n* = 509)	2004	2010	p value
PF	(+)→(+)	51.2 ± 9.4	45.8 ± 10.0	0.2135
	(+)→(-)	52.6 ±7.8	47.9 ± 11.0	0.0392
	(-)→(+)	51.3 ± 11.4	45.8 ± 13.6(5)	0.0225
	(-)→(-)	53.8 ± 8.4	52.5± 8.9(5)	0.0085
RP	(+)→(+)	45.9 ± 11.6	42.6 ± 9.5	0.594
	(+)→(-)	49.6 ± 10.2	49.4 ± 9.7	0.9032
	(-)→(+)	49.5 ±10.3	46.0 ± 10.1(3)	0.264
	(-)→(-)	51.9 ± 8.5	50.2 ± 9.7(3)	0.1368
BP	(+)→(+)	46.0 ± 8.9	42.2 ± 6.0(1)	0.5147
	(+)→(-)	45.4 ± 9.7	47.1 ± 8.1(1)	0.3458
	(-)→(+)	42.1 ± 7.7	45.3 ± 8.3(4)	0.4802
	(-)→(-)	52.7 ± 9.3	52.0 ± 9.4(4)	0.4522
GH	(+)→(+)	48.2± 8.3	44.5 ± 8.5	0.0929
	(+)→(-)	49.1 ± 9.5	48.0 ± 6.4	0.9255
	(-)→(+)	50.4 ± 8.0(1)	48.7 ± 7.9(2)	0.2569
	(-)→(-)	52.6 ± 8.9(1)	51.8 ± 9.5(2)	0.314
VT	(+)→(+)	48.7± 7.3	47.0 ± 6.8	0.7671
	(+)→(-)	49.0 ± 10.3	49.4 ± 8.8	0.7089
	(-)→(+)	50.3 ± 11.0	50.4 ± 8.0	0.8101
	(-)→(-)	55.1 ± 8.0	51.8 ± 8.7	<0.0001
SF	(+)→(+)	48.5 ± 13.6	46.9 ± 9.8	0.2604
	(+)→(-)	50.4 ± 8.3	48.3 ±10.4	0.1442
	(-)→(+)	48.9 ± 11.7	49.0± 8.9	0.4254
	(-)→(-)	51.8 ± 8.9	50.5 ± 9.2	0.1663
RE	(+)→(+)	49.4 ± 11.9	44.9 ± 7.0	0.3743
	(+)→(-)	49.0 ± 11.0	48.7 ± 10.3	0.7825
	(-)→(+)	46.8 ± 12.7	46.4 ± 10.6(2)	0.7982
	(-)→(-)	52.2 ± 7.9	50.8 ± 9.1(2)	0.5126
MH	(+)→(+)	47.5 ± 3.0	46.1 ± 12.3	0.6165
	(+)→(-)	46.4 ± 10.2	48.4 ± 9.7	0.3252
	(-)→(+)	49.2 ± 8.9	49.1 ± 9.8	0.9711
	(-)→(-)	50.4 ± 9.3	51.7 ± 8.5	0.0381

Average ± SD (1) $p < 0.05$, (2) $p < 0.01$, (3) $p < 0.005$, (4) $p < 0.001$, (5) $p<0.0001$

Abbreviations: LSS, lumbar spinal stenosis; SF-36, The 36-Item Short Form Health Survey; SD, standard deviation; PF, physical functioning; RP, role-physical; BP, bodily pain; GH, general health perception; VT, vitality; SF, social functioning; RE, role-emotional; MH, mental health.

Table S4. Predictive factors for LSS-symptom presence at the end of six-year follow-up period; Subjects without either knee or hip OA in 2004

		OR	95%CI	p value
Gender	Female	0.787	0.417–1.485	0.4597
Age	40–49 y		Reference	
	50–59 y	1.628	0.381–6.954	0.5107
	60–69 y	1.593	0.420–6.044	0.4935
	70–79 y	2.558	0.677–9.668	0.1661
BMI	< 18.5		Reference	
	18.5–25.0	1.297	0.353–4.764	0.695
	25.1–30.0	0.788	0.197–3.163	0.7373
	30 <	3.631×10^{-6}	–	0.9756
LSS symptoms	Positive	2.438	1.126–5.280	0.0238
RDQ score (Norm-based)	< 50	2.421	1.171–5.006	0.0171
Knee OA	Positive	–	–	–
Hip OA	Positive	–	–	–
Comorbidities	Respiratory	4.618×10^{-6}	–	0.9883
	Diabetes Mellitus	0.613	0.122–3.082	0.5521
	Cardiovascular	2.658	1.077–6.561	0.0339
	Cerebrovascular	3.021	0.131–69.394	0.4893
	Hypertension	1.104	0.596–2.044	0.7538
Smoking	Pack-Year ≥ 20	1.374	0.715–2.639	0.3406
Depressive symptoms	None		Reference	
	Mild	1.129	0.475–2.682	0.7834
	Moderate	0.883	0.305–2.555	0.8181
	Severe	1.168	0.425–3.208	0.763

Abbreviations: LSS, lumbar spinal stenosis; BMI, body mass index; RDQ, Roland-Morris Disability Questionnaire; OA, osteoarthritis

Table S5. Risk ratio of leg symptoms resulting in LSS development during the six-year follow-up period; Subjects without either knee or hip OA in 2004

Initial analysis		Development of LSS at six-year follow-up	
		LSS (+)	LSS (-)
Leg symptoms in 2004	(+)	50	135
	(-)	4	252

Abbreviations: LSS, lumbar spinal stenosis

Table S6. Predictive factors of the operation for LSS during the six-year follow-up period; Subjects without either knee or hip OA in 2004

		OR	95%CI	p value
Gender	Female	1.023	0.252–4.147	0.9746
Age	40–49 y			
	50–59 y	0.187	0.015–2.381	0.1963
	60–69 y	0.166	0.021–1.293	0.0864
	70–79 y	0.392	0.054–2.833	0.4289
BMI	< 18.5		Reference	
	18.5–25.0	9.534×10^{6}	–	0.9892
	25.1–30.0	1.449×10^{6}	–	0.9889
	30 <	2.058	–	0.9997
LSS symptoms	Positive	3.159	0.617–16.182	0.1676
RDQ score (Norm-based)	<50	0.683	0.107–4.362	0.6872
Knee OA	Positive	–	–	–
Hip OA	Positive	–	–	–
Comorbidities	Respiratory	2.490×10^{-6}	–	0.997
	Diabetes Mellitus	1.647×10^{-6}	–	0.9908
	Cardiovascular	1.034	0.119–8.975	0.9757
	Cerebrovascular	9.103×10^{-6}	–	0.9973
	Hypertension	1.553	0.401–6.018	0.5243
Smoking	Pack-Year ≥ 20	1.579	0.376–6.632	0.5328
Depressive symptoms	None		Reference	
	Mild	4.04	0.886–18.417	0.0828
	Moderate	2.42×10^{-6}	–	0.9859
	Severe	2.598	0.432–15.616	0.3808

Abbreviations: LSS, lumbar spinal stenosis; BMI, body mass index; RDQ, Roland-Morris Disability Questionnaire; OA, osteoarthritis
